# Graded Brittle–Ductile Transition via Laser-Induced Thermal Gradient for Broaching of Z10C13 Steel

**DOI:** 10.3390/mi17020204

**Published:** 2026-02-02

**Authors:** Guozhen Liu, Zhen Meng, Junqiang Zheng, Weiguang Liu, Xinghua Wu, Jing Ni, Haohan Zhang

**Affiliations:** School of Mechanical Engineering, Hangzhou Dianzi University, Hangzhou 310018, China; 231010011@hdu.edu.cn (G.L.); mengzhen@hdu.edu.cn (Z.M.); zhengjunqiang@hdu.edu.cn (J.Z.); 232010128@hdu.edu.cn (W.L.); 232010052@hdu.edu.cn (X.W.); nj2000@hdu.edu.cn (J.N.)

**Keywords:** martensitic stainless steel, Z10C13, laser surface processing, broaching performance, gradient modification, skin effect

## Abstract

This paper presents a breakthrough in activating the skin effect at conventional broaching speeds (1–8 m/min) by using laser defocus gradient modification to induce surface embrittlement in martensitic stainless steel Z10C13. Through controlled defocusing, a 50 μm gradient remelting layer was created, which features ultrafine grains (0.8 μm) and a high-density geometrically necessary dislocation (GND) zone (ρGND = 2.27 μm^−3^). The quasi-cleavage fracture was triggered via dislocation pinning by non-oriented low-angle grain boundaries (28.4% LAGBs). Multiscale characterization confirms that this microstructural transformation enhances surface hardness by 12.95% (reaching 31.4 HRC), reduces cutting force by 34.07%, and improves surface roughness by 63.74% (Sz = 28.80 μm). Simultaneously, a parallel crack-deflection mechanism restricts subsurface damage propagation, resulting in a crack-free subsurface zone. These results demonstrate the effectiveness of the embrittlement–toughening dichotomy for precision machining of difficult-to-cut materials under low-speed constraints.

## 1. Introduction

A series of difficult-to-cut materials has gained increasing utilization as engineered component surfaces in aerospace, nuclear power, and precision manufacturing industries due to their exceptional hardness and wear resistance [1,2]. Martensitic stainless steel Z10C13, as a representative difficult-to-machine material, exhibits superior corrosion resistance against most chemical media while maintaining outstanding mechanical properties including tensile strength, yield strength, toughness, and plasticity [3,4]. Broaching, as the main means of processing difficult-to-cut materials, can achieve high efficiency and high-quality machining of Z10C13 [5]. During the broaching processes, however, the material’s high hardness combined with poor chip breakability significantly compromises the surface integrity through accelerated tool wear and surface imperfections such as burrs and micro-spalling, particularly during the precision machining of hardened phases [6,7,8]. These challenges originate principally from dynamic work hardening mechanisms induced by thermomechanical coupling effects [9], where transient cutting forces interact synergistically with evolving residual stresses and microcrack propagation, fundamentally constraining machining precision while promoting concurrent surface damage accumulation and rapid tool degradation [10,11].

The laser-assisted machining strategy reduces cutting resistance and dynamically adjusts the work hardening behavior by inducing local thermal effects [12]. Moreover, laser heat treatment is achieved by depositing controlled energy from a high-power density laser beam onto the surface of the material [13]. The process involves rapidly heating the surface by photon absorption, followed by self-quenching by heat conduction to the substrate [14]. This thermal cycling induces phase transitions (e.g., austenite to martensitic), which enables the need for rapid surface hardening [15]. However, it is important to clarify that conventional laser heat treatment processes, such as laser quenching and annealing, primarily aim to achieve uniform hardness improvement or softening within a predetermined depth. Their through-thickness thermal input often leads to an expanded heat-affected zone, making it difficult to precisely control the gradients in microstructure and properties, and may even induce undesired degradation of the substrate properties. Currently, there remains a research gap regarding in-depth mechanisms and improvement strategies to address these inherent limitations. Therefore, there is an urgent need to conduct research on the precise control of laser-assisted machining processes to optimize surface integrity and suppress the initiation and propagation of near-surface damage [16].

Generally, the processing of martensitic stainless steel involves adjusting various process parameters to enhance machining performance and the quality of the workpiece. These adjustments can include reducing the cutting layer thickness and feed rate at lower speeds, increasing the rake angle to sharpen the cutting edge, and reducing the friction between the tool face and the chip base [17]. They can also include utilizing cutting fluids with excellent lubricating properties to minimize friction, artificially heating the cutting zone such as through electric hot machining, and using carbide tools for high-speed cutting with reduced rake angles to increase cutting temperatures [18]. In the case of steel cutting, achieving temperatures of up to 500 °C can help prevent the formation of scale marks. It has not solved the machining problems of the difficult-to-cut materials [19].

In practical machining processes, complex correlations between multiple process parameters and critical characteristics often complicate the balance between machining efficiency and quality [20]. A novel approach involves applying the “skin effect” to material damage under high-strain, high-strain-rate conditions; high-strain-rate-induced embrittlement can be achieved by increasing the cutting speed and decreasing the depth of cuts, effectively reducing machining forces and enhancing surface integrity in high-speed machining processes. The material deformation at high strain rates (>10^3^ s^−1^) leads to embrittlement, which in turn contributes to the skin effect of subsurface damage, resulting in better machined surface quality. Such high strain rates are typically triggered at high cutting speeds (5000 m/min) [21]. During the cutting process of martensitic stainless steel, a hardened layer is generated due to deformation, leading to a gradual increase in cutting force. High cutting speeds promote plastic deformation, further increasing the cutting force, which causes instability in the cutting process, reduces machining quality, affects the service life of tools and workpieces, and leads to an increase in cutting temperature, thereby influencing the machined surface quality [22,23,24,25]. Therefore, the “skin effect” induced by high speeds does not apply to the machining of martensitic stainless steel. However, this method demands cutting speeds frequently unattainable under operational constraints, particularly in precision broaching where velocities typically range from 1 to 8 m/min and even lower for ultra-precision applications [26]. Such low-speed conditions prevent adequate thermomechanical activation required for effective material embrittlement via the skin effect. These subcritical regimes not only disable crack suppression mechanisms but also amplify subsurface crack propagation through extended tool–workpiece contact durations [27].

This study demonstrates the manifestation of skin effect during low-speed cutting processes following laser surface treatment [21]. Contrasting with conventional techniques focused exclusively on parameter optimization, this work introduces a novel approach that synergistically enhances material machinability and surface quality through laser-induced skin effect activation. Significantly, we establish that high-speed machining is not a prerequisite for achieving skin effect in metal cutting, thereby creating a new paradigm for efficiency and quality enhancement in parameter-constrained operations like broaching. The research systematically investigates laser-pre-treated composite cutting processes through the comprehensive analysis of Z10C13, proposing an innovative gradient-modified layer design achieved through laser parameter modulation to realize graded distributions of grain size/orientation [28,29]. The uniqueness of the proposed “laser-induced surface embrittlement transformation” strategy stems from its use of high-energy-density lasers to rapidly melt and resolidify the material, creating a functional surface layer tens of micrometers thick with a graded mechanical property profile. The primary goal of this strategy is to achieve a controlled embrittlement within this surface layer to enhance cutting performance. This is accomplished alongside a minimal heat-affected zone, a result of the extremely rapid cooling rate, which effectively preserves the intrinsic toughness of the underlying substrate [30]. The study quantitatively resolves the influence of laser irradiation on crystalline nucleation dynamics and elucidates the competitive mechanism between the remelted layer and the altered layer during laser surface modification. Analysis confirms that the designed gradient treatment provides a unique materials science solution. This architecture progresses from a superfine-grained brittle surface layer through a transition zone to the unaffected ductile substrate, successfully reconciling the typically conflicting objectives of low cutting force, high surface integrity, and low subsurface damage. The approach thus transcends the traditional paradigm of merely pursuing surface hardening. The research further clarifies the mechanistic basis by which laser pre-treatment regulates machinability indices, cutting force characteristics, and surface integrity. A key breakthrough is the development of gradient-structured modified layers, which enable controlled thermomechanical responses through spatially tuned laser processing parameters [31,32,33].

## 2. Experiment and Method

### 2.1. Materials and Tools

The experimental cutting tool utilizes YG8 cemented carbide (Co = 8 wt% and WC = 92 wt%) in a single-tooth broach configuration, procured from Ma’anshan Cemented Carbide Cutting Tools Co., Ltd. (Maanshan, China). This material demonstrates significant advantages as the tool substrate owing to its high strength and wear resistance. The mechanical properties of YG8 are detailed in Table 1.

Z10C13, a hardenable stainless steel category, achieves property optimization through thermal processing via quenching and tempering treatments. The experimental material consists of Z10C13 with a nominal hardness of HB210 and density of 7.80 g/cm^3^. Its chemical composition, comprising primarily iron (Fe), carbon (C), chromium (Cr), and manganese (Mn) as major constituents, is detailed in Table 2.

### 2.2. Experiment

The experimental study aimed to systematically investigate how laser processing parameters affect the machinability and surface quality of Z10C13, while assessing machining efficiency and tool wear characteristics. Surface modification was conducted using a Han’s Laser IPC-K20-CS fiber laser marking system (Han’s Laser Technology Industry Group Co., Ltd., Shenzhen, China). In the experiments, the over-focus distance was considered as an input process parameter. The selected laser processing parameters are presented in Table 3 below.

Prior to determining the formal experimental parameters, this study first conducted systematic preliminary experiments aimed at establishing a qualitative relationship between the defocus distance and the surface modification effect. A series of single-factor experiments were performed across a broad defocus distance range (e.g., from −6 mm to +12 mm). Through macroscopic morphology observation, optical microscopy, and microhardness measurement, the surface state (presence or absence of melting, oxide color), modified layer depth, and hardness trends under different parameters were rapidly assessed.

Based on these preliminary test results, three representative nodes with distinct effects were selected: Group OF-0 (Δf = 0 mm) serves as the critical point producing significant melting/solidification and the strongest hardening effect; Group OF-10 (Δf = 10 mm) serves as the far endpoint that just avoids surface melting but induces a noticeable thermal effect and softening trend; and Group OF-5 (Δf = 5 mm) serves as a clear transitional point between the two aforementioned extreme effects. These three points are not precise theoretical extrema but were experimentally screened as key representatives of process windows that can reliably and repeatedly exhibit three typical laser–material interaction modes: “deep melting and quenching”, “transitional mixing”, and “shallow annealing”. This selection aims to maximally reveal the influence of defocus distance (energy density) on the properties of the modified layer and the subsequent cutting performance, as well as to identify the transition thresholds involved.

The divergence of the laser beam causes a progressive increase in spot radius with rising over-focus distance:

(1) $$d=d_{0}+2\Delta f\tan\left(\theta \right)$$

Laser spot area:

(2) $$A=\pi {\left(\frac{d}{2}\right)}^{2}$$

Power density:

(3) $$I=\frac{P}{A}$$

Dwell time:

(4) $$t=\frac{d}{t_{es}}$$

3D heat transfer model:

(5) $$\Delta T=\frac{\alpha Id}{\kappa }\sqrt{\frac{t}{\pi a}}$$

where *d* represents the actual spot diameter, $d_{0}$ is the ideal spot diameter, Δ*f* is the over-focus distance, *A* is the spot area (μm^2^), d is the spot diameter (μm), I is the laser power density (W/m^2^), *P* is the laser power (W), *t* is the dwell time (s), tes is the etching speed (s), Δ*T* is the temperature rise (*κ*), α is the thermal diffusivity (m^2^/s), *κ* is the thermal conductivity (W/(m·K)). With reference to the ASM Handbook, the thermal conductivity (*κ*) of Z10C13 at high temperatures is approximately 26.8 W/(m·K), the thermal diffusivity (α) is approximately 6.5 × 10^−8^ m^2^/s, and the specific heat capacity (Cp) is approximately 460 J/(kg·K).

Theoretical calculations indicate that both OF-0 and OF-5 groups (Δ*T* ≈ 2860 °C) significantly exceed the melting point of Z10C13. In practice, the actual temperature is moderated by the latent heat of melting, while vaporization-induced plasma formation creates an energy barrier that limits laser energy absorption, thereby stabilizing the surface temperature near the melting point. By comparison, the OF-10 group exhibits a theoretical temperature rise of 1144 °C, approaching but not exceeding the material’s melting point [34].

**Figure 1:**
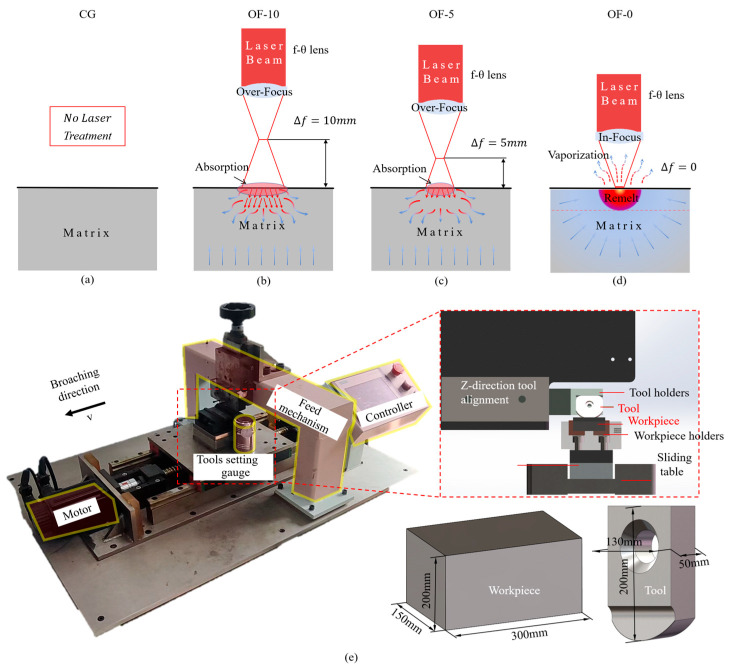
(**a**–**d**) Principle of laser processing about the different focus distances. (**e**) Broaching test site layout.

Figure 1a–d shows the laser principle and the corresponding sample grouping for different laser parameters. Figure 1a: CG was treated without laser treatment as a control group. Figure 1b: Group OF-10 corresponds to an off-focus distance of =10 mm. Figure 1c: Group OF-5 corresponds to an off-focus distance of =5 mm. Figure 1d: Group OF-0 represents the on-focus condition, with the off-focus distance set to Δf = 0 mm. Figure 1e shows the physical diagram of the homemade single-tooth broaching workstation, with the red dashed box enlarging the specific positional relationship and structure of the tool–workpiece–machine. The sample is a block type, and the tool has a single-tooth curved edge. The depth of cut is assured using a tool setting gauge before broaching.

Then, the hardness test of each group of samples after laser treatment was carried out to obtain the hardness change in the material surface after laser treatment. Vickers hardness measurements were performed on surfaces across all material groups using a 1 kg (9.807 N) test load and a 10 s dwell time according to ASTM E92 [35], averaging three points at each sampling location.

Following the experimental protocol, broaching tests were performed using a three-dimensional force sensor (KISTLER9119AA2, Jiangsu Donghua Test Technology Co., Ltd., Taizhou, China) with a sampling frequency of 1024 Hz to record cutting forces. Post-processing examinations employed a high-speed digital camera (KEYENCE VW-9000, KEYENCE Corp., Osaka, Japan) to analyze chip and workpiece morphologies.

Comparative experiments between laser-modified and untreated workpieces were conducted on a single-tooth broaching testbed, with the experimental configuration illustrated in Figure 1 and detailed parameters provided in Table 4.

Subsequently, the surface morphology of the machined specimens was characterized using a Sensofar S neox 3D non-contact optical profilometer (Sensofar S neox 3D, Sensofar S.L., Terrassa, Spain). The examination of the tool’s cutting edge (tool tip) was performed using a Keyence VW-9000 depth-of-field optical microscope. A 50× objective lens was employed during image acquisition.

## 3. Results

### 3.1. Laser Treatment Surface

#### 3.1.1. Laser-Induced Affected Layer

**Figure 2:**
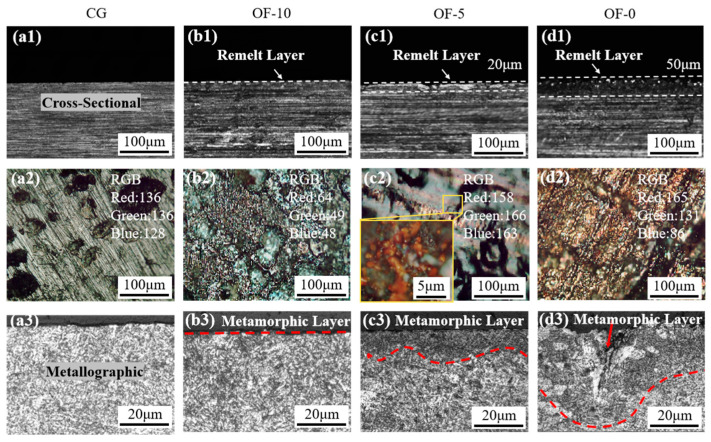
Material cross-sections and surface morphology under different laser conditions. (**a1**–**d1**) Optical images of cross-sections showing remelted layers under varying laser conditions. (**a2**–**d2**) Surface micrographs depicting oxidation and surface textures. (**a3**–**d3**) Metallographic images of cross-sections showing modified layers and ablation features.

As shown in Figure 2, optical microscopic images of the surface and cross-section of the samples under different laser conditions are presented. When the material surface was treated with different lasers’ over-focus distances, different depths of the modified layer were found, mainly due to the different laser pulse energy densities causing different degrees of heat-affected zones (HAZs). Particularly, compared with other samples, the OF-0 group material surface had the most intense reaction, showing the deepest remelted layer (see Figure 2(d1)), forming a 50 μm region from the cross-section, and the OF-0 sample surface obtained a laser-scanned groove with a gold surface free of oxides (see Figure 2(d2)). With the increase in the over-focus distance, the reaction on the sample surface became milder and the depth of the remelted layer decreased. In Figure 2(b1,c1), the OF-5 sample formed a 20 μm deep remelted layer, and the remelted layer of the OF-10 sample was the shallowest and almost invisible. In Figure 2(b2,c2), the rough black surface of the OF-10 sample indicated a slow oxidation forming an incomplete film, while the smooth black surface of the OF-5 sample with deep brown oxide reflection accelerated the reaction. The appearance of the remelted layer on the sample surface was due to the rapid absorption of high-density pulsed laser energy by the material surface, causing the material surface to melt. At the same time, due to the rapid self-cooling effect of the metal, the surface of the workpiece rapidly solidified again, producing a quenching effect similar to that in [36]. However, with the increase in the over-focus distance, the laser spot area on the material surface became larger and the energy density was lower, resulting in a larger heat-affected zone. At this time, the sample was subjected to an effect similar to annealing.

Metallographic experiments were conducted on the cross-section of the samples to obtain the metallographic images of the cross-section. In Figure 2(a3), uniform and regular plate-like martensite grain bundles can be observed. With the decrease in the over-focus distance, the metamorphic layer in the metallographic images of the sample cross-section (see Figure 2(b3–d3)) became deeper. In these figures, the red dotted line delineates the boundary between the metamorphic layer and the unaffected substrate material. The OF-0 sample had the deepest modified layer and local ablation pits. The surface layer transforms into acicular or lens-shaped lamellar martensite (see Figure 2(d3)). This was because part of the material on the surface of the OF-0 sample absorbed a large amount of pulsed laser beam energy and sublimated. Complete phase transformation (including martensitic transformation) was limited to the metamorphic layer, and this microstructure change directly controlled the machinability and cutting mechanism of the material.

**Figure 3:**
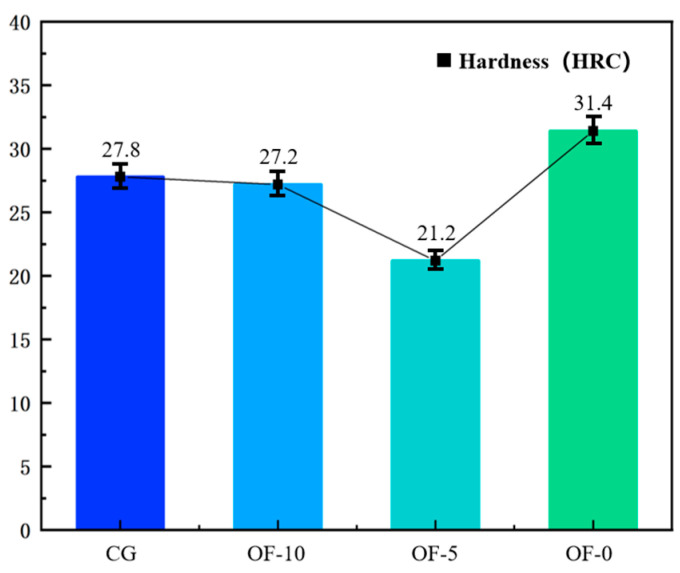
The average value of the workpiece surface hardness measurement.

As shown in Figure 3, the surface hardness of the samples has undergone significant changes after being processed with different parameters. It can be observed that as the laser over-focus distance decreases, the surface hardness of the samples first decreases and then increases. This is mainly due to the change in laser energy density. Generally speaking, in the heat-affected zone, traditional laser heat treatment causes softening through local microstructure recrystallization and expands the softened area through heat conduction. The surface hardness of CG is 27.8 HRC, while the surface hardness of the OF-10 and OF-5 groups decreased by 2.16% and 23.74%, respectively. However, in the OF-0 group, the highly concentrated pulsed laser energy triggered abnormal hardening through extreme thermal cycling, increasing the hardness by 12.95%. A detailed analysis will be presented in the specific conclusion.

#### 3.1.2. EBSD Analysis

**Figure 4:**
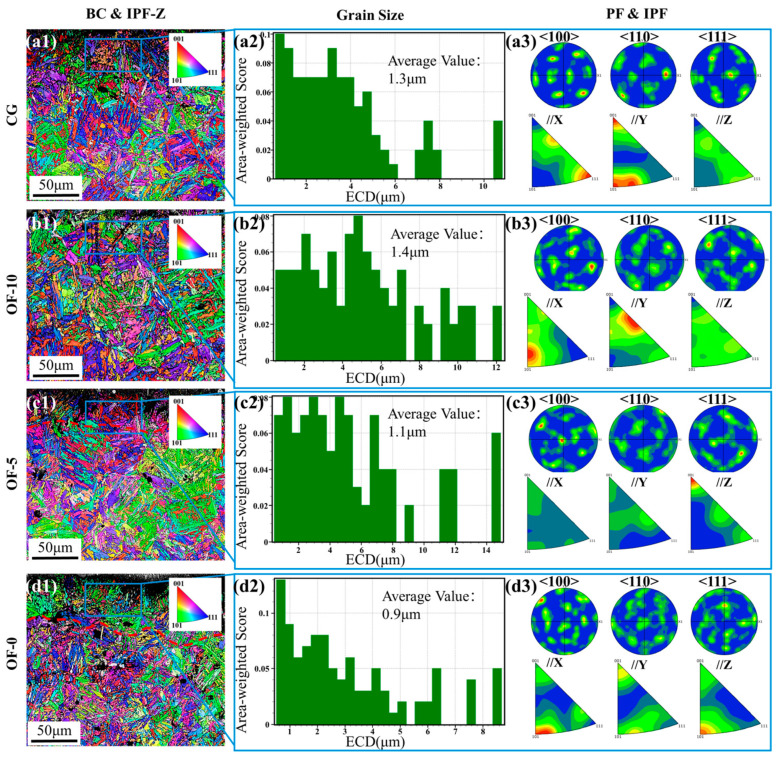
EBSD analysis of material microstructure under different laser conditions. (**a**) CG group: (**a1**) BC map and IPF map along the *Z*-axis; (**a2**) grain size distribution within the red box in (**a1**); (**a3**) pole figures and inverse pole figure within the red box in (**a1**). (**b**) OF-10, (**c**) OF-5, and (**d**) OF-0 groups: (**b1**,**c1**,**d1**) BC and IPF maps; (**b2**,**c2**,**d2**) grain size distributions; (**b3**,**c3**,**d3**) pole figures and inverse pole figures.

To further elucidate the hardening mechanisms, electron backscatter diffraction (EBSD) imaging of the near-surface regions was performed with step sizes tailored to each group’s grain structure to ensure optimal spatial resolution. As shown in Figure 4(a1–d1), Martensite laths show color zoning in the IPF-Z map, where laths of the same color belong to the same orientation. If adjacent martensite grains have uniform colors, it indicates that they have a very small orientation difference. BC and IPF-Z grain boundary analysis reveals significant surface refinement in the OF-5 and OF-0 groups (see the red dashed lines in Figure 4(c1–d1)), with the OF-0 group demonstrating the deepest refinement layer. Among them, the OF-0 group shows the deepest refinement layer, and the surface layer of its samples forms needle-like martensite due to laser-induced phase transformation, and dense twins can be seen in the microstructure.

ASTM/ISO standard grain measurements (blue-framed regions in Figure 4(a2–d2)) confirm the smallest average grain size (30.77% reduction compared to CG group) in the OF-0 group, 15.38% in the OF-5 group, and 7.69% in the OF-10 group. Pole figures and inverse pole figures (see Figure 4(a3,d3)) further demonstrate that the CG group exhibits localized <110> concentration with overall dispersion, imparting high toughness anisotropy; the OF-0 group develops extreme <111>//Z texture; the OF-10 group shows uniform <111> distribution coupled with strong Z-direction texture, optimizing machinability; the OF-5 group features <100> concentration coupled with <110>/<111> dispersion, enhancing toughness but inducing stress concentration [37].

**Figure 5:**
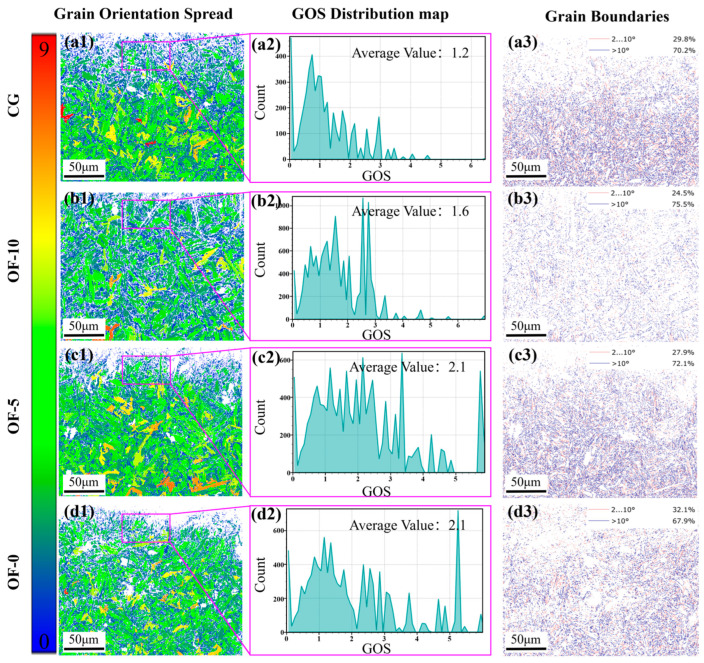
EBSD analysis of grain orientation and boundaries under different laser conditions. (**a**) CG group: (**a1**) GOS map; (**a2**) GOS distribution within the red box in (**a1**); (**a3**) grain boundaries map. (**b**) OF-10, (**c**) OF-5, and (**d**) OF-0 groups: (**b1**,**c1**,**d1**) GOS maps; (**b2**,**c2**,**d2**) GOS distributions within the red boxes in (**b1**,**c1**,**d1**); (**b3**,**c3**,**d3**) grain boundaries maps.

The intrinsic {110}<111> dominant slip system of the BCT-structured matrix material is modulated by laser over-focus through energy density-controlled microstructural evolution. High-energy input in the OF-0 group triggers quenching to form ultrafine grains (0.9 μm), with dislocation slip proliferation causing a sharp increase in low-angle grain boundaries (see Figure 4 and Figure 5); simultaneously, steep thermal gradients along the melt pool’s vertical direction drive <001> orientation epitaxial growth (see Figure 4(d3)), while GPa-level thermal stress activates the {110}<111> slip system, inducing crystal rotation under Schmid factor control to form a strong <111>//Z texture. Grain refinement and texture synergistically increase hardness by 12.95% and optimize the processing efficiency, though restricted dislocation motion and slip system constraints reduce plastic accommodation capacity. In contrast, low-energy input in the OF-10/OF-5 groups induces annealing effects where grain coarsening and texture randomization balance toughness but exacerbate the stress concentration, fundamentally resulting from energy density polarization: high density drives non-equilibrium quenching, while low density promotes thermal coarsening [38].

To deepen the study of laser pulse-induced changes in surface machinability, an in-depth analysis of the material’s grain orientation propagation (GOS) plot was performed to assess the plastic deformation capacity. From Figure 5(a1–d1), a significant difference in grain orientation uniformity can be observed in the GOS plot, and the CG group serves as the control group; its GOS plot (Figure 5(a1)) shows a large area of concentrated color patches. Conversely, OF-0 shows a uniform small area of color patches, which indicates significant grain refinement and a concentration of geometrically necessary dislocations (GNDs) in the high GOS region.

Figure 5(d2) is the GOS distribution map of the purple boxed area (remelted layer on the surface of the material after laser treatment), and the average value of GOS in the OF-0 group is 2.1. Compared to the CG group’s GOS mean of 1.2 (Figure 5(a2)), both OF-0 and OF-5 increased by 75% (Figure 5(d2,c2)), and OF-10 increased by about 33% (Figure 5(b2)). Combined with the grain boundaries plot of Figure 5(a3–d3), the LAGB was 29.8% in the CG group, 24.5% in the OF-10 group, and 27.9% in the OF-10 group. The OF-0 group had the highest LAGB of 32.1%. This indicates that there is serious lattice distortion and energy accumulation in the OF-0 group, and the dense dislocation tangles hinder the coordinated slip. At the same time, the dynamic recrystallization inhibited at high cooling rates limits the sub-grain boundary shape (GOS ≈ 0.5–1.5°) and instead retains the high GOS region that promotes microcrack nucleation along the low-angle boundary. The increase in small-angle grain boundaries (2–10°) in OF-0 is due to the rearrangement of dislocations to sub-grain boundaries during dynamic recovery, and indicates that sub-grains coalesce to high-angle grain boundaries (>10°), which migrate to the consuming high-strain region. This increases the local plasticity of the surface layer of the material, making the local brittle transformation possible.

In addition, it can be found that with the decrease in laser focus distance, the proportion of large-area color patches and red patches (highly distorted lattices) in the GOS map gradually decreases, and the proportion of GOS mean and LAGB increases. This is because with the decrease in the laser eccentric distance, the spot shrinks, and the increase in laser energy density leads to a sharp increase in the temperature gradient and strain gradient, which induces the evolution of grain boundaries, which in turn induces the GND-mediated sub-crystallization process, hinders the migration of HAGB, and leads to an increased risk of cracking along the sub-grain boundary.

In summary, the performance trade-off of OF-0 is regulated by energy density, especially Hall–Petch strengthening and brittleness transition. The high-density input maximizes GND-mediated strain localization but inhibits recrystallization-driven toughness recovery [39].

**Figure 6:**
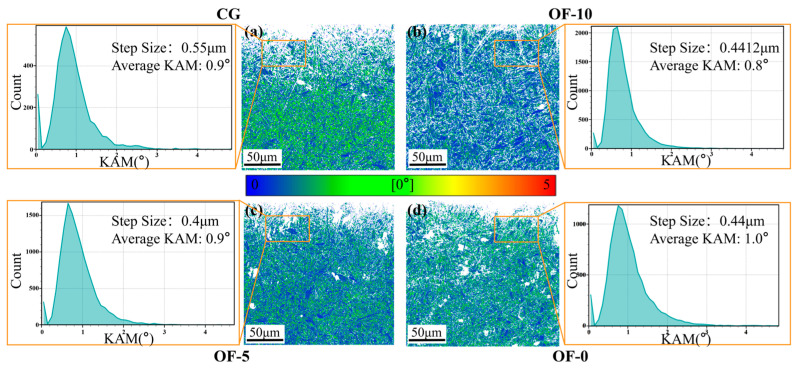
KAM analysis under different laser conditions. (**a**) CG. (**b**) OF-10. (**c**) OF-5. (**d**) OF-0.

Figure 6 presents kernel average misorientation (KAM) diagrams characterizing local lattice distortion and strain distribution across material groups. Geometrically necessary dislocation (GND) densities were calculated using normalized step-size conversions:

Average KAM:

(6) $$KAM_{ave}=\exp\left[\frac{1}{N}\Sigma_{1}^{i}\ln{KAM}_{L,i}\right]$$

GND density:

(7) $$\rho^{GND}=2KAM_{ave}∕\mu b$$

where $KAM_{ave}$ represents the average KAM value of the selected area. ${KAM}_{L,i}$ is the local KAM value at point i. N is the number of points in the test area. μ is the step-size selected in the electron backscatter diffraction (EBSD) experiment, and b is the length of the Burgers vector. $\rho^{GND}$ ($\mu m^{-3}$) is the geometrically necessary dislocation density.

The GND of different samples was calculated through Equation (7). The GND density of CG was 1.64. Compared with CG, the GND density of OF-10 increased by 10.37%, and that of OF-5 increased by 37.20%. OF-0 had the highest increase of 38.41% in GND density, which was 2.27. This was mainly attributed to the dislocation slip at the material surface and the dislocation locking in the rapid solidification zone of the molten pool. Additionally, it could be observed that the GND density showed an increasing trend due to the reduction in the lasering distance. This indicates that the increase in laser energy density is the fundamental cause of the increase in geometrically necessary dislocation (GND) density. With the increase in laser high energy density, the temperature gradient surges, leading to dislocation slip multiplication and plastic deformation. At the same time, the ultra-high cooling rate causes the occurrence of martensitic transformation, resulting in a nonlinear enhancement of the thermomechanical phase transformation coupling effect.

These findings demonstrate that the OF-0 group’s performance advantages originate from synergistic interactions between the ultrafine grains and strong <111> texture. The refined grain structure enhances strength by impeding dislocation motion through increased boundary density, while the pronounced texture restricts slip systems to elevate hardness. However, this orientation singularity concurrently reduces material toughness.

Although grain refinement-induced work hardening and dislocation accumulation improve hardness via Hall–Petch strengthening, excessive dislocation tangles in OF-0 hinder coordinated deformation, promoting brittleness. High-KAM regions further accelerate brittle fracture through crack nucleation. This makes it possible to reduce cutting energy consumption.

### 3.2. Cutting Performance

#### 3.2.1. Cutting Result

**Figure 7:**
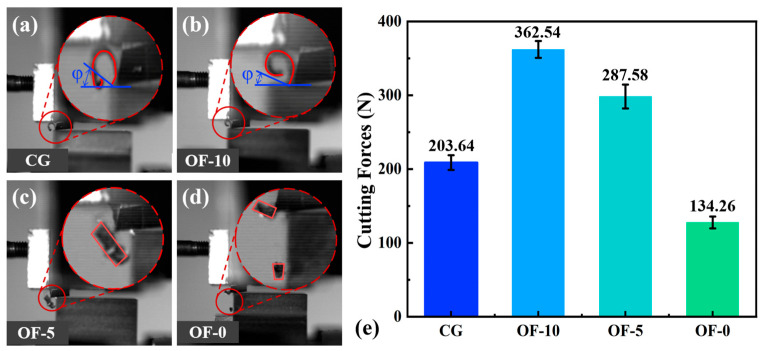
(**a**–**d**) Chip morphology during cutting process captured by high-speed camera and cutting forces. (**e**) Comparison of cutting forces across conditions.

From Figure 7a–d, it can be seen that both the CG group and the OF-10 group were performing ductile cutting, producing continuous chips. However, in the OF-10 group, due to increased plasticity, the chip curl radius and shear angle (φ) significantly decreased. The OF-5 group formed fragmented particles through localized brittle fracture (Figure 7c). The OF-0 group showed a clear brittle fracture mechanism and demonstrated the best machining efficiency, with the minimum cutting force. This is directly evidenced by the microstructural evolution. First, laser thermal shock induces high-density dislocation tangles and nano-precipitates (KAM implied by elevated GOS = 1.8, Figure 5(d2)), strongly impeding slip system activation (dislocation pinning strengthening). This refines grains (small-area GOS blocks, Figure 5(d1)) and elevates boundary density (32.1% LAGBs, Figure 5(d3)), restricting dislocation motion but eliminating plastic accommodation capacity.

**Figure 8:**
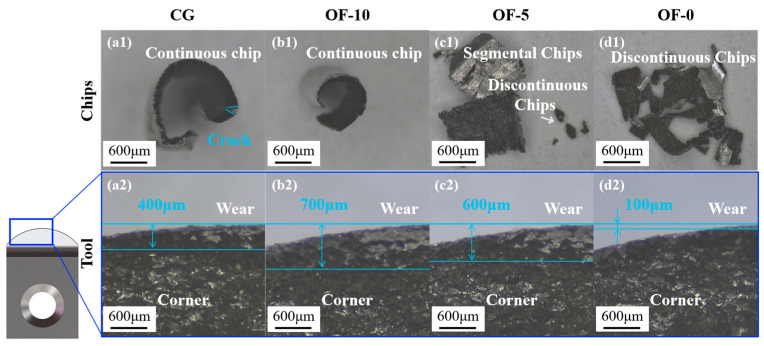
Chip shape and tool wear. (**a1**–**d1**) represent the chip morphology of different groups. (**a2**–**d2**) represent the wear conditions of tools in different groups.

Concurrently, localized remelting triggers grain boundary embrittlement: elemental segregation reduces grain boundary cohesion, lowering crack propagation resistance. Combined with strain concentration in machining, this promotes cleavage fracture (discontinuous chips, Figure 7d) and intergranular cracking (high-GOS zones, Figure 5(d1)). Crucially, reduced plastic energy dissipation concentrates the fracture energy into brittle pathways, explaining both low cutting force fluctuations (Figure 7e) and suppressed tool damage (Figure 8). Thus, OF-0 achieves efficient material removal not despite but through controlled embrittlement: dislocation pinning and GB weakening synergistically minimize plastic dissipation, redirecting energy toward fracture-dominated chip separation.

After laser treatment, there were significant changes in the cutting force, as shown in Figure 7e. The cutting force for the CG group was 203.64, while the OF-10 group saw a 78.03% increase in cutting force, the OF-5 group increased by 41.22%, and the OF-0 group had a decrease of 34.07%. As the laser defocus distance decreased, the cutting force initially increased sharply and then dropped sharply. This counterintuitive phenomenon mainly results from a shift in the cutting mechanism. This is due to the increase in the microstructural changes in the surface material as the energy density of the laser pulses rises. The pinning at small angle boundaries intensifies work hardening (see Figure 5), while strain concentration in the non-recrystallized regions increases cutting resistance (Figure 6). Both the cutting forces of OF-5 and OF-10 increase sharply, which is consistent with their directional characteristics that elevate the risk of local stress concentration (see Figure 4). However, when the energy density surges beyond a certain level, the strain gradient spikes, causing partial remelting of the surface material, resulting in brittle fracture during cutting.

Figure 8(a1) clearly shows the front and back sides of a typical curled continuous chip: the back side exhibits a lamellar structure, while the front side is smooth and flat, indicating that these chips are produced by plastic deformation. Figure 8(d1), showing discontinuous chips, confirms the brittle fracture mechanism. This is mainly due to laser-induced microstructural changes that redirect energy to low-energy fracture paths, replacing ductile deformation (>100 μs chip formation) with cleavage-dominated fractures (<10 μs, Figure 7d) to suppress plastic dissipation and reduce cutting forces by 62% (see Figure 7e). This brittle transformation optimizes the grain boundary architecture, evidenced by GOS <1° (see Figure 5(d2)) and >60% HAGBs (see Figure 5(d3)), enabling intergranular fracture that converts continuous chip flow into brittle fragmentation while reducing tool wear through minimized tool–workpiece adhesion (see Figure 8). These properties originate from synergistic mechanisms: ultrafine grains (see Figure 5(d1)) and HAGBs maximize crack propagation efficiency; nanosecond-scale crack nucleation/extension enables flake-chip formation within 10 μs, demonstrating that strategic embrittlement—not ductility—drives machining breakthroughs via microstructural redesign that redirects plastic work to fracture work, achieving unprecedented efficiency–stability synergy.

Figure 8(a2–d2) is an optical image of the worn cutting edge, which is partially enlarged in the blue box selection of the tool. It can be observed that the trend of the wear depth is the same as that of the cutting force (Figure 7e), which is also a steep increase and then a steep decrease. From CG group 0.4 mm to OF-0 group there is a minimum wear depth of 0.1 mm. This is mainly due to the change in the microstructure of the surface of the material caused by different laser energy densities. The OF-10 group undergoes grain coarsening from laser heat absorption, where annealing softening enhances bulk toughness but generates continuous chips dominated by material ductility. Severe plastic deformation causes significant principal cutting force increases, exacerbating crater tool wear. As the laser defocus distance decreases, the elevated surface energy density induces instantaneous melting to form a remelted layer. The OF-0 group’s discontinuous chips from this brittle remelted layer suppress tool wear while maintaining low cutting forces (see Figure 7). In the OF-5 group, remelted layer thickness below the cutting depth allows the ductile substrate to promote segmented shear-zone chips while the brittle surface triggers localized discontinuous fracture, yielding a hybrid segmented–discontinuous chip morphology.

#### 3.2.2. Surface Quality

**Figure 9:**
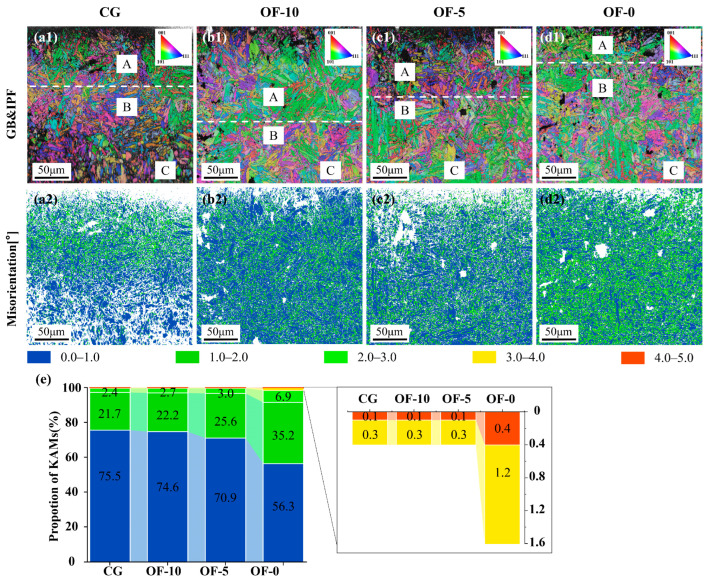
(**a1**–**d1**) EBSD analysis of the cross-sectional microstructure of a material after cutting, Zones A, B, and C represent areas from the machined surface to the substrate, categorized by the extent of lattice deformation. (**a2**–**d2**) The misorientation distribution. (**e**) The KAM values are distributed in proportions.

Inverse pole figure (IPF) color maps (Figure 9(a1–d1)) reveal severe crystallographic rotation near the machined surfaces (Zone A) with minimal lattice damage in bulk regions (Zone C), demonstrating expanded deformation zones and elevated shear energy dissipation under high-defocus-distance conditions. The OF-0 group exhibits substantially reduced Zone A dimensions and minimal shear energy consumption, directly explaining its lowest cutting forces (Figure 7).

**Figure 10:**
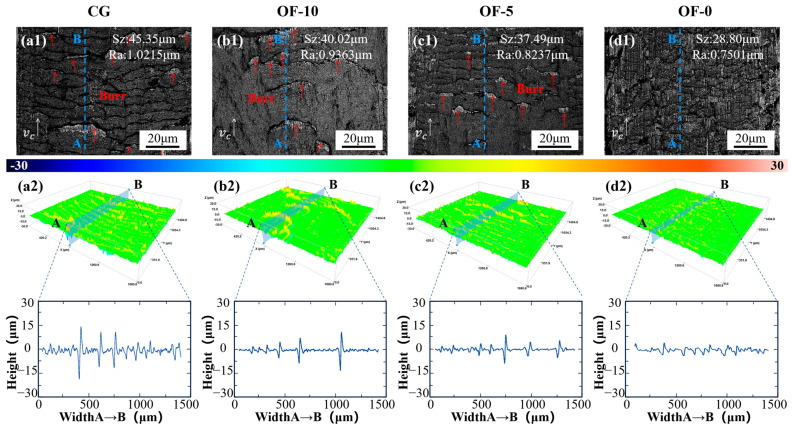
Analysis of machined surface topography and roughness under different laser conditions. (**a1**–**d1**) compare the burr morphology across different groups, where red arrows mark the burrs. (**a2**–**d2**) display the corresponding surface topography, showing in height fluctuation.

Figure 9(a2–d2) shows the misorientation distribution, and it can be observed that the proportion of blue patches (KAM value 0.0–1.0) decreases and the proportion of green patches (KAM value 1.0–2.0) increases as the laser focus distance decreases. To find out the proportion of less but higher KAM values, calculated as Σvalue (grain boundary)/total boundaries and displayed histographically (Figure 9e) [40], confirm OF-0′s predominant high-KAM regions indicating elevated dislocation density, intense lattice distortion, and significant residual stress fields. It reflects the higher degree of plastic deformation strain in the superficial layer of the machined surface. These microstructural features synergistically govern enhanced strength/hardness/wear resistance while improving fatigue performance through crack initiation suppression, ultimately yielding optimal surface quality (see Figure 10). As the defocus distance decreases, the depth of the subsurface plastic deformation zone induced by laser processing reduces, and this trend can also be quantified [41].

Under standard machining conditions, sharp burrs of different scales appear on the machined surface, and the burrs grow along the cutting direction, which are marked by red arrows in Figure 10(a1–d1). As the laser defocus distance decreases, the burr density gradually reduces, and the surface roughness also gradually decreases. White light interferometry (Figure 10(a2–d2)) shows stable Z-heights, with the height difference fluctuations exhibiting the same gradual decreasing tendency as the laser defocus distance decreases. For CG, Sz is 45.35 μm and Ra is 1.0215 μm. For OF-10, Sz is reduced by 11.75% and Ra by 8.34% compared to CG. For OF-5, Sz is reduced by 17.33% and Ra by 19.36% compared to CG.

Finally, the OF-0 group achieves the best surface quality, with Sz reduced by 36.49% and Ra by 26.57% compared to CG, and burrs are eliminated (Figure 10(d1)). This is mainly due to the homogenization of fine grains resulting from the high energy density of the OF-0 group, resulting in ultrafine grains (Figure 5(d1)), which enhance the material homogeneity and reduce cutting forces (Figure 7e). Reduced anisotropy inhibits tool vibration while the transition to brittle fractures prevents plastic pileup via intergranular cleavage (Figure 8). GOS homogenization (Figure 5(d2)), resulting from dislocation-assisted recrystallization and LAGB proliferation (Figure 5(d3)), elevates deformation resistance to eliminate burrs through micro-fracture redirection. These factors enable the 50 μm remelted zones to suppress damage via quasi-cleavage.

#### 3.2.3. Subsurface Damage

The minimal subsurface damage in the OF-0 group partially aligns with the “skin effect” mechanism, where high strain rates (>10^3^ s^−1^) induce material embrittlement to concentrate damage superficially [42]. However, this typically requires ultra-high-speed cutting (5000 m/min) for activation [21]. Here, laser modification achieves comparable outcomes. The OF-0 group exhibits a crack-free subsurface (Figure 10(d1)), non-directional LAGBs (28.4%), and minimal GND density (1.04 × 10^14^ m^−2^), confirming that brittle fracture replaces plastic deformation to form a protective surface layer. Crucially, high-speed cutting of martensitic stainless steel triggers work hardening that degrades stability and surface quality, rendering traditional skin effects inapplicable. Instead, the 50 μm remelted zone in OF-0 (Figure 5(d1)) promotes quasi-cleavage fractures via quenching and dislocation pinning (32.1% LAGBs, Figure 5(d3)), enabling subsurface protection at conventional speeds while reducing cutting forces (Figure 7e) and enhancing surface integrity (Ra = 0.7501 μm).

**Figure 11:**
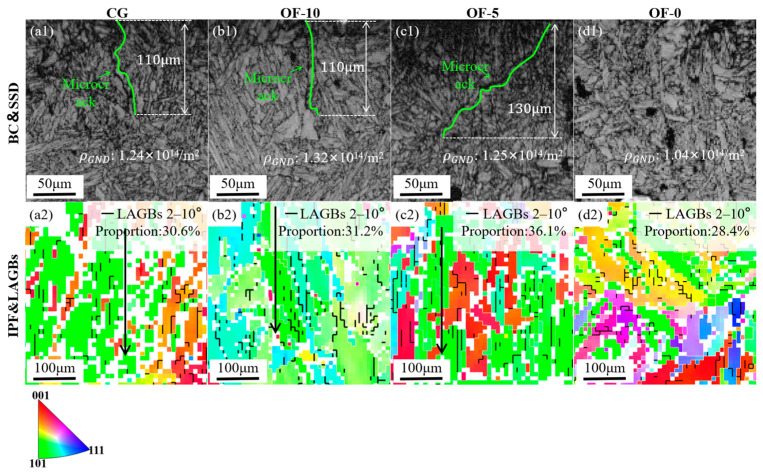
Crack and LAGBs distribution. (**a1**–**d1**) display microcrack morphologies for each group. (**a2**–**d2**) illustrate the distribution of low-angle grain boundaries (LAGBs), with arrows denoting the preferential orientation of grain deformation and reconstruction.

Contrastingly, CG and OF-10 exhibit vertical microcracks with a crack depth of 110 μm and GND densities of 1.24 × 10^14^ m^−2^ and 1.32 × 10^14^ m^−2^, respectively. LAGBs have a vertical arrangement, 30.6% and 31.2%, respectively, where boundary dislocation pileup ignites intergranular cracking. OF-5 shows a 45° inclined crack with a crack depth of 130 μm, a GND density of 1.25 × 10^14^ m^−2^, and a LAGB of 36.1%, indicating shear-dominant damage. Figure 10(a2–c2) reveals LAGBs oriented perpendicular to the machined surface along grain boundaries, elevating intergranular cracking risks and exacerbating subsurface damage. Conversely, the non-directional LAGBs in OF-0 (28.4%, Figure 11(d2)) eliminate preferential crack paths, synergizing with minimal GND density (1.04 × 10^14^ m^−2^) to suppress damage.

## 4. Discussion

**Figure 12:**
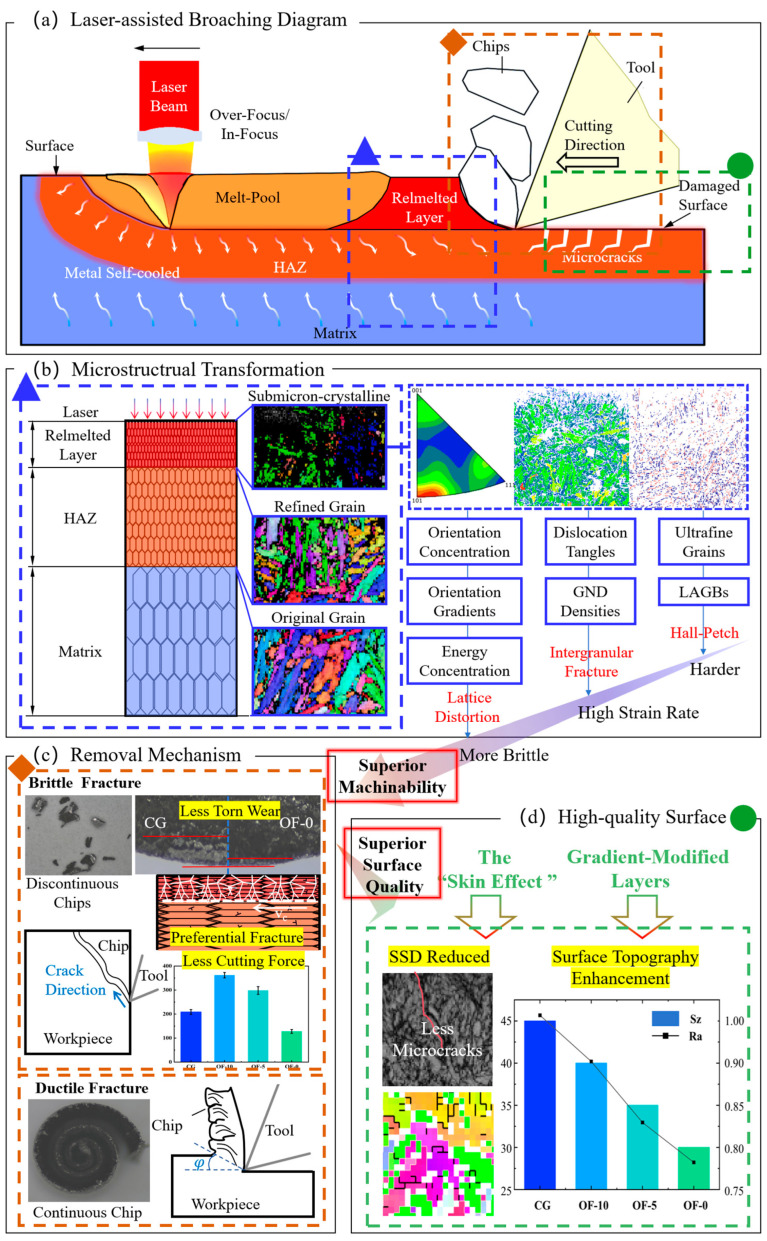
Laser-assisted high-efficiency and high-quality processing mechanism. (**a**) shows the Laser-assisted Broaching Diagram, depicting the synergistic action of the laser and the cutting tool. (**b**) presents the underlying Microstructural Transformation induced by laser thermal shock. (**c**) details the Removal Mechanism of material under the coupled thermo-mechanical effect. (**d**) the formation mechanism of the High-quality Surface.

Figure 12 illustrates the principle of laser-assisted skin effect activation and efficient high-quality processing for Z10C13. The overall laser-assisted broaching process is shown in Figure 12a, where laser treatment forms a remelted layer and a deeper HAZ on the material surface. Due to the metal self-cooling effect, a strong temperature gradient develops between the surface layer and the substrate. This gradient fundamentally alters the material microstructure, as detailed in the blue-boxed section of Figure 12b marked with a triangle symbol. Specifically, high-energy laser pulses drive four key evolutions in the remelted layer (Figure 2): crystal orientation redistribution (Figure 4), lattice distortion (Figure 5), dislocation reorganization (Figure 6), and ultimately enhanced macroscopic processing performance.

Crucially, by controlling laser defocus to concentrate high-density energy in the removal layer, this process constructs a stratified architecture with divergent fracture mechanisms. The remelting layer gradually becomes brittle, while the deeper heat-affected zone (HAZ) is only affected by temperature and gains toughness enhancement. As depicted in the orange-boxed section of Figure 12c (rhombic symbol), the above treatment method constructs a layered structure with completely different fracture mechanisms. Smaller cutting forces (Figure 7) and reduced tool wear (Figure 8) are thereby achieved through rapid crack propagation that generates discontinuous chips, significantly improving processing efficiency.

The green-boxed analysis in Figure 12c (rotundity symbol) confirms subsurface damage suppression. Property differences between the embrittled surface and toughened HAZ inhibit crack propagation into the substrate. Concurrently, reducing the laser spot size concentrates the energy density, transforming single-pulse thermal cycles from deep/uniform (OF-10, OF-5) to shallow/steep (OF-0). This generates extreme surface properties that activate the skin effect—redirecting crack paths (Figure 11), minimizing subsurface damage, and enabling simultaneous high surface quality and processing efficiency despite the material–property dichotomy.

The superior machining performance of the OF-0 stems from laser-induced microstructural transformations that fundamentally reconfigure the material behavior. High-energy-density laser processing generates a 50 μm gradient-modified remelted layer characterized by ultrafine grains (0.8 μm), elevated dislocation density (ρGND = 2.27 μm^−3^), and strong <111> crystallographic texture. These modifications collectively enhance surface brittleness through Hall–Petch strengthening and slip system restriction, enabling quasi-cleavage fracture initiation at conventional cutting speeds (1–8 m/min). The brittle-dominant material removal mechanism replaces energy-intensive plastic deformation, evidenced by flake-type chips and 63.74% cutting force reduction, while strain-rate sensitivity activates a low-speed “skin effect” that confines damage to the superficial layer. Crucially, non-directional low-angle grain boundaries (28.4% LAGBs) deflect microcracks and suppress subsurface damage propagation. Under high laser energy density (OF-0), a 50 μm remelted layer forms (Figure 5(d1)), where the ultrafine grain structure (Figure 5(d1)) enhances material isotropy and suppresses tool vibration. Through quenching hardening and dislocation pinning (32.1% LAGBs, Figure 5(d3)), this configuration actively constrains plastic flow, triggering quasi-cleavage fracture that intercepts plastic accumulation (built-up edge elimination, Figure 10(d1)) and mitigates cutting force fluctuations. This synergistic effect actively eliminates scale thorns and achieves a surface roughness of Sz = 28.80 μm, representing a 63.74% improvement over conventional processing. This tripartite mechanism, which includes grain refinement, fracture control, and damage confinement, overcomes a critical limitation. Laser-generated gradient layers bypass the high-speed requirement for skin effect activation through fundamentally transforming deformation physics.

## 5. Conclusions

This study proposes a new laser-assisted processing strategy for Z10C13, which utilizes high-energy laser pulses to modify the surface layer of Z10C13, thereby achieving high-quality machining during broaching. A systematic analysis of the surface layer microstructure evolution behavior of the material under different parameters was conducted, revealing the skin effect at conventional cutting speeds. The main findings show that

(1) Laser parameters precisely regulate surface transformation by controlling the defocus distance to concentrate high-density energy, generating a 50 μm gradient-modified remelted layer with ultrafine grains (0.8 μm) and an elevated dislocation density (ρGND = 2.27 μm^−3^). EBSD-confirmed microstructural transitions result directly from temperature gradient-induced rapid solidification.
(2) The OF-0 group consequently achieves a 63.74% surface roughness reduction and minimized cutting forces via brittle-dominant removal, where dislocation pinning at non-directional low-angle grain boundaries (28.4% LAGBs) actively deflects microcracks to confine subsurface damage.
(3) The laser-modified layer enables low-speed skin effect through dual mechanisms: strain-rate sensitivity coupled with the sharp embrittlement–toughening transition between the surface and substrate. Cutting stress multiplies dislocations to locally embrittle the surface, while the abrupt property transition at the remelted layer/HAZ interface deflects cracks toward the surface, constraining the damage to a shallow depth and generating discontinuous chips. This approach overcomes the traditional speed limitation for achieving skin effect in martensitic stainless steel.

## Figures and Tables

**Table 1:** Mechanical properties of the YG8 (%).

Density	Impact Energy	Hardness	Bending Strength
g/cm^3^	J/cm^2^	HRA	MPa
14.6–14.9	2.5	89	1500

**Table 2:** Chemical composition of Z10C13 stainless steel (%).

C	Si	Mn	P	S	Cr	Ni
0.15	1	1	0.035	0.03	13.5	0.6

**Table 3:** Laser processing parameters.

Parameter	Laser Wavelength	Output Power	Spot Diameter	Repetition Frequency	Etching Speed	Repetition Accuracy
Unit	μm	W	μm	kHz	mm/s	μm
Value	1.06	20	30	20–200	500	±3

**Table 4:** Single-tooth broaching conditions for orthogonal experiment.

Samples		Broaching Variables		
		Over-Focus Distance Δf (mm)	Cutting Depth $a_{p}$ (mm)	Cutting Speed $v_{c}$ (mm/s)
Control group	CG	No laser treatment	0.05	40
Experimental group	OF-10	+10	0.05	40
	OF-5	+5	0.05	40
	OF-0	0 (in-focus)	0.05	40

## Data Availability

The raw data supporting the conclusions of this article will be made available by the authors on request.

## Abbreviations

The following abbreviations are used in this manuscript:

ASTM	American Society for Testing and Materials
ISO	International Organization for Standardization
BC Map	Band Contrast Map
BCT	Body-Centered Tetragonal
EBSD	Electron Backscatter Diffraction
GND	Geometrically Necessary Dislocation
GOS	Grain Orientation Spread
HAGBs	High-Angle Grain Boundaries
LAGBs	Low-Angle Grain Boundaries
HAZ	Heat-Affected Zone
IPF	Inverse Pole Figure
KAM	Kernel Average Misorientation
